# Impact of Glucagon-Like Peptide 1 Receptor Agonists on Biochemical Markers of the Initiation of Atherosclerotic Process

**DOI:** 10.3390/ijms25031854

**Published:** 2024-02-03

**Authors:** Marcin Hachuła, Michał Kosowski, Sabina Ryl, Marcin Basiak, Bogusław Okopień

**Affiliations:** 1 Department of Internal Medicine and Clinical Pharmacology, Medical University of Silesia, Medyków 18, 40-752 Katowice, Poland; marcin.hachula@gmail.com (M.H.); mkosowski@sum.edu.pl (M.K.);; 2 Department of Anaesthesiology and Intensive Care, Municipal Hospital in Zabrze-Biskupice, Zamkowa 4, 41-803 Zabrze, Poland; rylsabina@gmail.com

**Keywords:** GLP-1, semaglutide, dulaglutide, Il-1, TNFα, oxLDL, atherosclerosis, atherosclerotic plaque

## Abstract

Atherosclerosis stands out as one of the leading causes of global mortality. The inflammatory response against vascular wall components plays a pivotal role in the atherogenic process. The initiation of this process is notably driven by oxidized low-density lipoprotein (oxLDL) and a range of pro-inflammatory cytokines, with interleukin-1β (Il-1β) and tumor necrosis factor α (TNFα) emerging as particularly significant in the early stages of atherosclerotic plaque formation. In recent years, researchers worldwide have been diligently exploring innovative therapeutic approaches for metabolic diseases, recognizing their impact on the atherogenesis process. Our study aimed to investigate the influence of glucagon-like peptide 1 receptor agonists (GLP-1RA) on cytokine concentrations associated with the initiation of atherosclerotic plaque formation in a group of patients with type 2 diabetes and dyslipidemia. The study encompassed 50 subjects aged 41–81 (mean: 60.7), all diagnosed with type 2 diabetes, dyslipidemia and confirmed atherosclerosis based on B-mode ultrasound. Following a 180-day treatment with dulaglutide or semaglutide, we observed a statistically significant reduction in biochemical markers (oxLDL, TNFα and Il-1β) associated with the initiation of the atherosclerotic process (*p* < 0.001) within our study group. In addition to the already acknowledged positive effects of GLP-1RA on the metabolic parameters of treated patients, these drugs demonstrated a notable reduction in proinflammatory cytokine concentrations and may constitute an important element of therapy aimed at reducing cardiovascular risk.

## 1. Introduction

Atherosclerosis and its complications have remained one of the main causes of death in the world for many years [1]. For this reason, in recent years, scientists around the world have been trying to develop modern forms of therapy for metabolic diseases, the development of which results in the intensification of the atherogenic process.

The process of atherogenesis itself is closely reminiscent of an autoimmune disease, because the inflammatory reaction against the components of the vascular wall plays a key role in it [2]. One of the most important roles in initiating this process is played by oxidized low-density lipoprotein (oxLDL) particles, which are recognized by T lymphocytes located in the vascular wall [3]. In response to a foreign antigen, they trigger an immune reaction dependent on T lymphocytes, leading to a gradual intensification of the inflammatory process within the blood vessel wall, which ultimately leads to further damage to it and an intensification of the inflammatory response that is the basis for the atherosclerotic plaque formation process [4].

The immune system reaction discussed above involves a whole range of pro-inflammatory cytokines produced not only by activated cells of the immune system but also by damaged cells of the vascular wall [5]. Among all of them, interleukin 1β (Il-1β) and tumor necrosis factor α (TNFα) seem to be the most important in the initial stages of the formation of atherosclerotic plaque.

Il-1β is a representative of the entire Il-1 family with pro-inflammatory effects [6]. Its activation within the blood vessel wall is related to the activation of the nucleotide-binding oligomerization domain-like receptor, pyrin domain-containing 3 (NLRP3) inflammasome, by accumulating cholesterol crystals [7]. Activating the inflammasome in this way ultimately leads to the activation of caspase 1, which transforms pro-Il-1β into its active form [8]. The active form of Il-1β is responsible for the progression of atherosclerotic lesions, as indicated by many clinical studies, proving that its higher concentration is associated with a greater risk of death in patients suffering from heart failure [9] and patients after a myocardial infarction [10]. What seems even more important is that targeted therapy with canakinumab, an Il-1β inhibitor, resulted in a reduction in the risk of death from cardiovascular causes in the group of patients with diagnosed coronary artery disease [11].

TNFα is another cytokine that is very important in the development of atherosclerotic plaques. It belongs to the TNF ligand superfamily, and it is synthesized as a type II integral membrane protein occurring in a vast number of cell types. Within the vascular wall, its action is strongly related to oxidative stress [12]. Oxidative stress induced by this cytokine is a known factor in increasing the risk of cardiovascular diseases. Another important phenomenon is necrosis or apoptosis of cardiomyocytes induced by high concentrations of TNFα [13].

However, it is worth noting that, apart from the elevated concentration of low-density lipoprotein (LDL) cholesterol, other diseases are also responsible for intensifying the inflammatory process, which in themselves constitute additional risk factors for cardiovascular events. Such diseases include obesity [14] and type 2 diabetes [15].

This is why the attention of scientists around the world is focused on a group of drugs called glucagon-like peptide 1 receptor agonists (GLP-1RA). Due to their beneficial effect on reducing body weight and improving glycemic control, they also seem to significantly influence the development of atherosclerotic plaques in blood vessels, which has been confirmed in animal models [16]. Several large randomized clinical trials such as LEADER, PIONEER, SUSTAIN and REWIND have shown a reduction in cardiovascular risk in patients with diabetes treated with GLP-1RA. [17,18,19,20]. For this reason, the European Society of Cardiology Guidelines for the management of cardiovascular disease in patients with diabetes have listed these as the preferred drugs in the group of diabetic patients at high risk of cardiovascular events [21]. Unfortunately, there are still no studies clearly indicating the mechanism by which this group of drugs significantly reduces cardiovascular risk.

The aim of our study was to investigate the effect of GLP-1RA on the concentrations of cytokines involved in the process of initiating the formation of atherosclerotic plaque in a group of patients with type 2 diabetes and dyslipidemia.

## 2. Results

### 2.1. Study Group Characteristics

The study group consisted of 50 patients with a mean age of 60.76 ± 9,52 years, including 27 women and 23 men. All subjects were diagnosed with type 2 diabetes mellitus (median glycated hemoglobin (HbA1c): 8.75%; the average duration was 10 years) and dyslipidemia. The concomitant diseases included hypertension (80%), chronic kidney disease (18%, all in stage G3a), hypothyroidism (16%) and heart failure with reduced ejection fraction (8%). Treatment was not changed during the intervention. Therefore, our control group included 26 sex-matched healthy subjects.

Whole baseline characteristics and a comparison of the metabolic parameters between the study and control groups are presented in Table 1 and Table 2.

### 2.2. Metabolic Effect after 180 Days of Treatment

In the study group after treatment, we observed a statistically significant reduction in anthropometric parameters like body mass index (BMI) (*p* < 0.001). Also, we observed a statistically significant decrease in blood pressure (systolic blood pressure (SBP) and diastolic blood pressure (DBP)-*p* < 0.001). In biochemical tests, we observed a lower concentration of fasting glucose and HbA1c (mean: 7.56% average reduction of 1.12%; *p* < 0.001). In terms of lipid profile, we did not notice any statistically significant changes. More importantly, we observed a statistically significant decrease in the fibrosis-4 score (FIB-4) (*p* < 0.001). Detailed results are presented in Table 3.

### 2.3. Effect on Biochemical Markers of Initiation of Atherosclerotic Process

**Figure 1:**
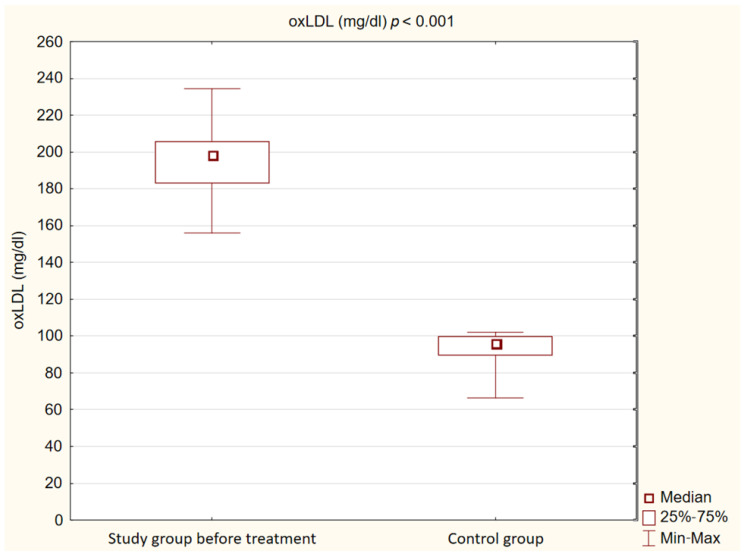
Concentration of oxidized low-density lipoprotein (oxLDL) in study and control groups.

**Figure 2:**
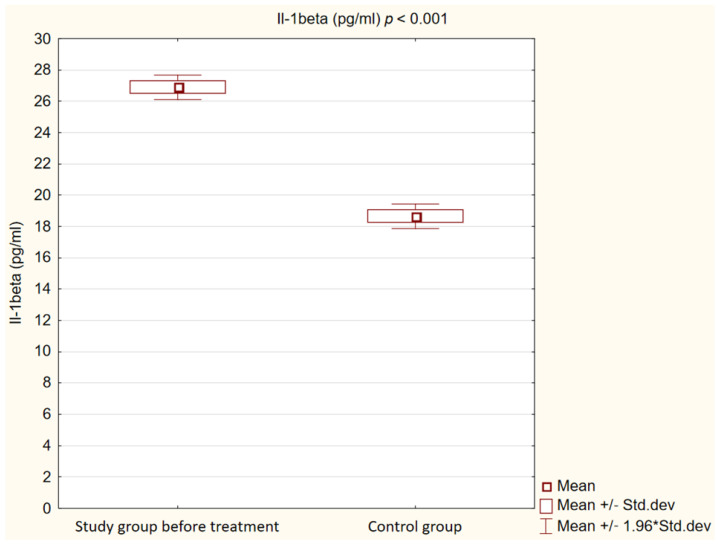
Concentration of interleukin 1β (IL-1beta) in study and control groups.

**Figure 3:**
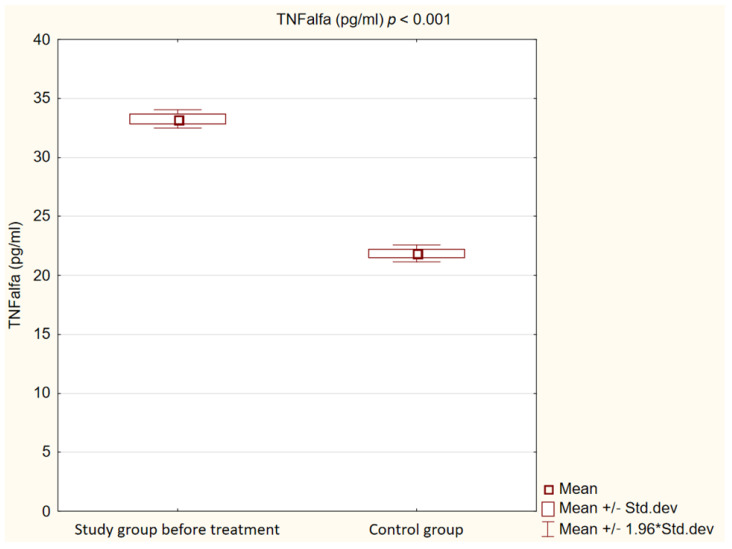
Concentration of tumor necrosis factor α (TNFalfa) in study and control groups.

We observed that in the control group, concentrations of Il-1β, TNFα and oxLDL were statistically significantly lower (*p* < 0.001) than in the study group before treatment. Detailed results are presented in Figure 1, Figure 2 and Figure 3.

**Figure 4:**
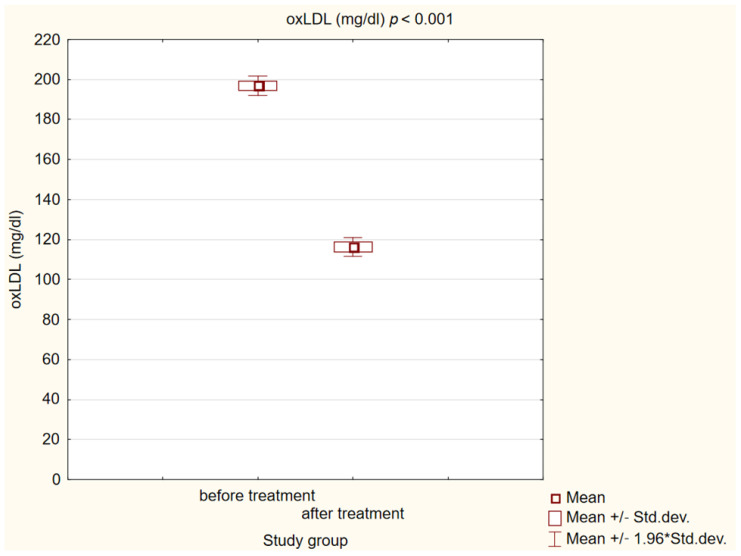
Concentration of oxidized low-density lipoprotein (oxLDL) in study group before and after treatment.

**Figure 5:**
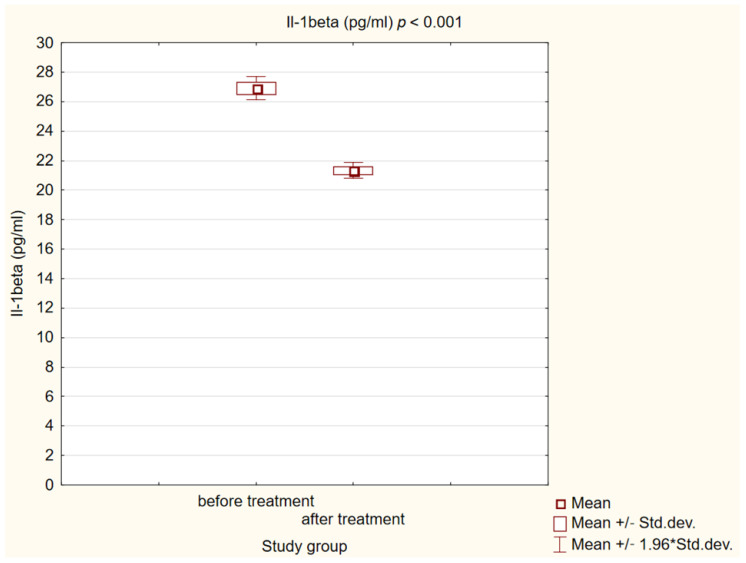
Concentration of interleukin 1β (Il-1beta) in study group before and after treatment.

**Figure 6:**
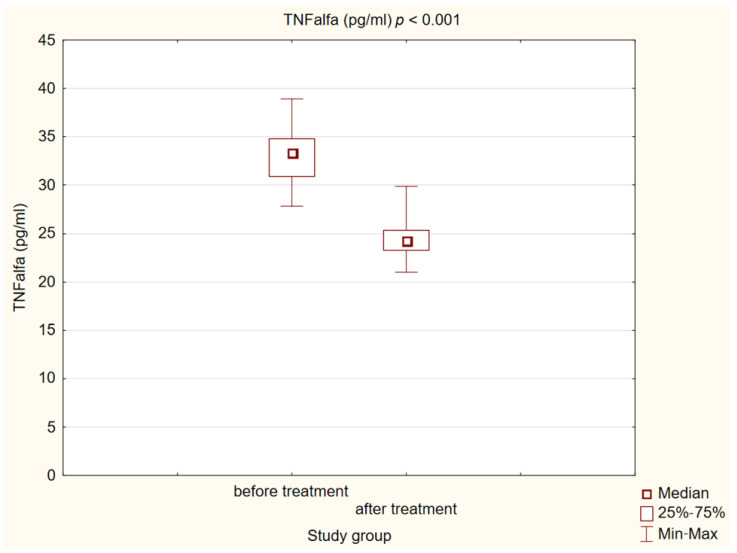
Concentration of tumor necrosis factor α (TNFalfa) in study group before and after treatment.

After 180 days of treatment, we also observed a statistically significant decrease in biochemical markers of initiation of the atherosclerotic process (*p* < 0.001) in our study group. The results are presented in Figure 4, Figure 5 and Figure 6.

An even more important observation we made is the fact that changes in the concentrations of pro-inflammatory cytokines do not correlate in any way with changes in HbA1C concentrations and body weight, which indicates that GLP-1RA, independent of the effect on metabolic parameters, leads to inhibition of the inflammation in our body. Detailed information on the correlations we examined is presented in Table 4.

### 2.4. Safety and Adverse Events

Thirty-one patients (62%) reported adverse effects. They mostly concerned the gastrointestinal system: 28% reported a feeling of fullness, 32% reported nausea and 22% reported diarrhea. No serious adverse events were reported during the study. Only 12% of patients described the above-mentioned side effects as impairing normal functioning. Two patients discontinued therapy due to adverse events.

## 3. Discussion

Our study clearly proves that GLP-1RA, in addition to its beneficial effect on the patient’s metabolic profile, reduces the concentration of cytokines responsible for initiating the formation of atherosclerotic plaque. This phenomenon may be one of the reasons for the reduction in cardiovascular risk induced by these drugs, which has been observed in clinical trials.

This effect has been studied very extensively in relation to other groups of drugs used in lipid-lowering treatment, which, thanks to their pleiotropic anti-inflammatory effect, were supposed to further reduce cardiovascular risk. Statins and PCSK-9 inhibitors are particularly extensively studied drug groups in this context.

In accordance with statins, a large randomized clinical trial using atorvastatin appeared in 2011, which proved its significant impact in reducing the concentrations of pro-inflammatory interleukins such as interleukin 6 or Il-1β, which was also tested in our group of patients [22]. In 2022, a large meta-analysis of clinical trials also clearly indicated that statins significantly reduce TNFα concentrations [23]. The antioxidant effect of this drug group is also important, as it translates into lowering the concentration of oxLDL particles, as evidenced by a meta-analysis of 28 clinical studies prepared by Jamialahmadi T. et al. [24].

Researchers managed to observe similar effects in relation to one of the newest types of lipid-lowering therapy, i.e., therapy with PCSK-9 inhibitors. In the case of Il-1β, the first study appeared in May 2023, which proved the relationship between the use of evolocumab and the reduction in the concentration of these proinflammatory interleukins and inhibition of the inflammatory process [25]. With regard to TNFα, the first article confirming such a decrease in the concentration of this cytokine after the use of alirocumab was the work prepared by our team in 2022 [26]. There are currently no reports on the reduction in oxLDL concentration after the use of this group of drugs, but this year saw the publication of the first experimental work which may indicate a reduction in oxidative stress within cells after the use of PCSK-9 inhibitors [27].

At the moment, there are no studies that would compare the currently used lipid-lowering treatment with other groups of drugs that may have a beneficial effect on reducing cardiovascular risk, such as GLP-1RA.

As mentioned in the introduction, GLP-1RA undoubtedly reduces the risk of cardiovascular events, as evidenced by numerous clinical studies [17,18,19,20], but the mechanism of this risk reduction is not well known at this point. Taking into consideration that the cytokines we tested are involved in the process of creating the atherosclerotic plaque, the effect of this group of drugs we observed may be one of the reasons why GLP-1RA reduces the cardiovascular risk. However, additional large randomized clinical trials will be required to clearly determine this phenomenon.

The first report on the effect of liraglutide on reducing the concentration of pro-inflammatory cytokines was published in 2019. In this report, in an animal model, a decrease in the concentration of both pro-inflammatory cytokines we tested was observed after the use of this drug [28]. Similar results with response to Il-1β were obtained in an in vitro study in which the effect of exenatide on fibroblast-like synoviocytes isolated from patients suffering from rheumatoid arthritis was examined [29]. More importantly, another study in an animal model showed that exendin-4, by stimulating GLP-1R, protects beta-cells from Il-1β-induced apoptosis [30]. Several studies conducted in in vitro models also prove that the use of GLP-1RA reduces the uptake of oxLDL by monocytes, as well as the migration of monocytes to the vascular wall, which may ultimately slow down the development of atherosclerotic plaque [31,32,33,34].

There are still very few studies conducted on patients in this area. The only study that unquestionably indicates a beneficial effect on the downregulation of the inflammatory process in the vascular wall is the study conducted by Zobel E. et al. It shows that the use of liraglutide in patients reduces inflammation in the vessel, which was confirmed using a PET scan [35]. In accordance with the concentrations of pro-inflammatory cytokines also tested by our team, the same results were shown by Luna-Marco C. et al., whose study also indicates a decrease in TNFα concentration [36]. As for IL-1β, the only relevant scientific report was published in 2014, also indicating a beneficial effect of GLP-1RA treatment on the decrease in its concentration [37]. However, it should be mentioned that this study was conducted on a very small group of patients; it involved only 10 participants. In addition, several preclinical studies in mice with an experimental lung injury have shown that GLP-1RAs reduce the production of pro-inflammatory cytokines Il-1 or TNFalpha, among others. [38] So far, no studies in the literature have examined the effect of GLP-1RA on oxLDL concentration in patients treated with this group of drugs.

Our study has several limitations. Firstly, our study group included only 50 patients. Secondly, this study concerned only patients from the Upper Silesia region of Poland; the obtained results could be different due to location, race and environmental factors. Thirdly, while planning the study, we planned to use only semaglutide, but problems with supply and availability in the Polish market forced us to change the tested drug to dulaglutide during the study. The other important limitation of our study was the lack of a control group treated with a placebo or an active comparator.

## 4. Materials and Methods

### 4.1. Study Population

Out of 91 patients, we included 50 aged 41–81 (mean: 60.7) in the study. All subjects were diagnosed with type 2 diabetes, dyslipidemia and confirmed atherosclerosis based on B-mode ultrasound common carotid intima–media thickness. The medical experiment was performed in January 2022–September 2023. Individuals meeting all the highly specific and narrow inclusion and exclusion criteria were deemed eligible to participate in the study. Each patient gave their informed consent in accordance with the Declaration of Helsinki. All the information about the subjects was anonymized. Patients were recruited at the Department of Internal Medicine and Clinical Pharmacology in Katowice, Poland, and as referrals from the Mysłowice and Imielin diabetes outpatient departments. The study protocol was approved by the Bioethical Committee of the Medical University of Silesia PCN/CBN/0052/KB1/45/I/22.

All included subjects were treated with one of GLP-1RA, either semaglutide (*n* = 16) or dulaglutide (*n* = 34), at a typical hypoglycemic dose and administered every week at the same time of day. The choice of treatment was determined by the drugs’ availability on the Polish market. During the intervention, the GLP-1RA therapy was not modified. The intervention was conducted for a duration of 180 days.

### 4.2. Inclusion and Exclusion Criteria

The inclusion criteria were type 2 diabetes; dyslipidemia, defined as plasma total cholesterol (TC) > 200 mg/dL and/or triglycerides (TG) > 150 mg/dL, and the presence of atherosclerotic plaque in the common carotid artery, confirmed by ultrasound examination.

Patients were excluded from the study for the following reasons: type 1 diabetes; cardiac disorders, like the exacerbation of chronic heart failure and unstable coronary artery disease; or a history of percutaneous coronary intervention, coronary artery bypass grafting or stroke less than 3 months before starting the study. Other exclusion criteria included chronic pancreatitis; acute exacerbation of autoimmune disorders; pregnancy and breastfeeding; uncompensated thyroid disease; alcoholism; any acute and chronic inflammatory processes, including COVID-19 infection 4 weeks before inclusion in the study (acute infection is expressed as an increase in CRP values > 5 mg/dL) or leukocytosis; chronic kidney disease in a stage below G3b, with an estimated glomerular filtration rate (eGFR) < 45 mL/min/1.73 m^2^; acute and chronic liver diseases expressed as an increase in transaminases more than three times greater than the norm; or medical history of diagnosed chronic viral hepatitis. After the intervention, all of the subjects were interviewed once again. They were also excluded if, within the last 6 months, they increased their physical activity, changed their type of diet, their treatment was modified or they started therapy with a new drug with a proven effect on lipid serum levels or with a known pleiotropic effect (e.g., statins, fibrates, ezetimibe, niacin, non-selective beta-blockers, metformin, sodium–glucose cotransporter-2 (SGLT2) inhibitors, or ursodeoxycholic acid). They were also excluded if they had a cardiovascular event or suffered a severe infection.

### 4.3. Laboratory and Anthropometric Measurements

All measurements were taken before study enrollment and after 6 months of treatment by a physician. Body weight and height were measured before following standard procedures, and BMI was calculated in kg/m^2^. Waist and hip circumferences were measured at the typical location, and the waist/hip ratio (WHR) was computed. Arterial blood pressure was measured twice in the sitting position in the arm without vascular access. For this purpose, the Omron M400 Intelli IT automatic device was used. To estimate the eGFR, the CKD-EPI formula was used. The values were presented in mL/min/1.73 m^2^. Routine laboratory measurements were performed in the certificated laboratory, and venous blood samples were collected at 8 a.m. after 12 h of overnight fasting before the treatment and after 180 days of intervention. The plasma levels of cytokines were assessed by commercially available Enzyme-linked immunosorbent assay (ELISA) kits as described by the manufacturer for oxLDL (K7810, Immunediagnostik AG, Bensheim, Germany); Il-1β: (850.006.192, Diaclone, Besancon Cedex, France); TNFα: (950.090.192, Diaclone, Besancon Cedex, France).

### 4.4. Arteriosclerotic Plaque Examination

The examination of the carotid arteries and the assessment of complex intima–media thickness (C-IMT) in the extracranial segment was performed using B-mode ultrasound with a linear probe at a frequency of 7.5–10 MHz on a Hitachi Aloka F37 ultrasound machine. According to the Atherosclerosis Risk in Communities Study (ARIC), the C-IMT was evaluated 3 times and the average score was taken into account. The measurement was performed in the distal common carotid (1 cm proximal to the carotid bulb). For confirmation of atherosclerotic plaque in the carotid artery, we assumed a thickness of the C-IMT complex > 1.5 mm or the presence of plaque, in accordance with the guidelines.

### 4.5. Statistical Analysis

The data were processed using Statistica TIBCO Software Inc., Palo Alto, USA, (2017) version 13.3 software, which was licensed by the Medical University of Silesia in Katowice. To assess the normality of distributions, we used the Shapiro–Wilk test. Values are presented as means and 95% confidence intervals or medians with Q1–Q3 values. During the preparation of the baseline characteristics of the study and control groups, we compared groups by using χ^2^ and U Mann–Whitney tests. To compare quantitative variables, the *t*-test for independent means and *t*-test for dependent means were used. To compare independent variables with an abnormal distribution, the Mann–Whitney U test was used. For dependent variables, we used the Wilcoxon test. We also used Spearman’s rank correlation to assess the relationship between variables. We assumed a *p*-value of less than 0.05 was statistically significant.

## 5. Conclusions

In addition to the predetermined positive effect of GLP-1RA on the metabolic parameters of patients, these drugs significantly reduce the concentrations of IL-1β, TNFα and oxLDL, which are involved in initiation of the atherosclerotic process. Additional studies are needed to unequivocally assess the impact of this therapeutic method on the development of cardiovascular diseases and the risk of cardiovascular events.

## Figures and Tables

**Table 1:** Baseline characteristics of patients.

	Study Group	Control Group	*p*
Number of patients	50	26	
Age, years	60.76 ± 9.52	33.08 ± 5.45	<0.001
Women, %	54	50	0.74
Men, %	46	50	0.74
WHO guidelines on physical activity, %	40	54	<0.05
Smokers, %	18	4	<0.01
Alcohol abuse, %	0	0	1

**Table 2:** Comparison of metabolic parameters in the control and study groups. BMI—body mass index; WHR—waist/hip ratio; DBP—diastolic blood pressure; HbA1C—glycated hemoglobin; GFR—glomerular filtration rate; SBP—systolic blood pressure; TC—total cholesterol; LDL—low-density lipoprotein cholesterol; HDL—high-density lipoprotein cholesterol; non-HDL—non-high-density lipoprotein cholesterol; TG—triglycerides; FIB-4—fibrosis-4 score; SD—standard deviation; Q1—first quartile; Q3—third quartile after treatment.

	Study Group before Treatment			Control Group			
	Mean	SD		Mean	SD		* p *
BMI (kg/m^2^)	35.02	7.10		22.46	2.54		<0.001
WHR	0.97	0.06		0.83	0.09		<0.001
DBP (mmHg)	82.36	8.56		77.96	8.19		<0.001
	** Median **	** Q1 **	** Q3 **	** Median **	** Q1 **	** Q3 **	
SBP	135	125	145	122	120	132	<0.001
Glucose (mg/dL)	160.50	133.10	203.30	89	84.80	95	<0.001
TC (mg/dL)	166.55	150.30	199	173.80	156.10	196.60	0.72
LDL (mg/dL)	83	67	105	90.50	74	110	0.46
HDL (mg/dL)	48.95	43.70	56.80	64.30	58.80	71.10	<0.001
non-HDL (mg/dl)	113.70	100	155.60	109	86	129	0.15
TG (mg/dL)	167.95	110.50	207.10	86.50	62.30	108	<0.001
Creatinine (mg/dL)	1.06	0.98	1.15	0.93	0.79	1.03	<0.001
GFR (ml/min/1.73 m^2^)	67.50	58	81	96.50	86	113	<0.001
HbA1C (%)	8.75	7.60	9.60	5.50	5.20	5.70	<0.001
FIB-4	1.50	1.18	1.90	0.66	0.54	0.88	<0.001

**Table 3:** Effect of glucagon-like peptide-1 receptor agonists (GLP-1RA) on metabolic parameters. BMI—body mass index; WHR—waist/hip ratio; DBP—diastolic blood pressure; HbA1C—glycated hemoglobin; GFR—glomerular filtration rate; SBP—systolic blood pressure; TC—total cholesterol; LDL—low-density lipoprotein cholesterol; HDL—high-density lipoprotein cholesterol; non-HDL—non-high-density lipoprotein cholesterol; TG—triglycerides; FIB-4—fibrosis-4 score; SD—standard deviation; Q1—first quartile; Q3—third quartile after treatment.

	Study Group before Treatment			Study Group after Treatment			
	Mean	SD		Mean	SD		* p *
BMI (kg/m^2^)	35.02	7.10		33.21	6.94		<0.001
SBP (mmHg)	135.36	13.72		129.62	10.24		<0.001
DBP (mmHg)	82.36	8.56		78.76	5.62		<0.001
HbA1C (%)	8.68	1.52		7.56	1.04		<0.001
GFR (ml/min/1.73 m^2^)	69.76	14.51		73.10	18.21		0.11
	** Median **	** Q1 **	** Q3 **	** Median **	** Q1 **	** Q3 **	
WHR	0.96	0.92	1.02	0.96	0.92	1.01	0.13
Glucose (mg/dL)	160.50	133.10	203.30	138.30	117.40	164.10	<0.001
TC (mg/dL)	166.55	150.30	199	169.10	146	198	0.62
LDL (mg/dL)	83	67	105	80.50	60	117	0.53
HDL (mg/dL)	48.95	43.70	56.80	50.65	46.30	60.50	0.18
non-HDL (mg/dL)	113.70	100	155.60	107.80	93.40	148.20	0.19
TG (mg/dL)	167.95	110.50	207.10	146.50	104	189	0.28
Creatinine (mg/dL)	1.06	0.98	1.15	1.04	0.92	1.14	0.45
FIB-4	1.50	1.18	1.90	1.30	1.03	1.60	<0.001

**Table 4:** Correlation between changes in weight and concentration of glycated hemoglobin and concentrations of proinflammatory factors. HbA1C—glycated hemoglobin; Il-1β—interleukin 1β; TNFα—tumor necrosis factor α; oxLDL—oxidized low-density lipoprotein.

	ΔHbA1C	ΔWeight
ΔIl-1β	R = 0.012, *p* > 0.05	R = 0.015, *p* > 0.05
ΔTNFα	R = 0.039, *p* > 0.05	R = 0.001, *p* > 0.05
ΔoxLDL	R = 0.15, *p* > 0.01	R = 0.089, *p* > 0.05

## Data Availability

Data are contained within the article.

## References

[1] Rana J.S., Khan S.S., Lloyd-Jones D.M., Sidney S. (2021). Changes in mortality in Top 10 causes of death from 2011 to 2018. J. Gen. Int. Med..

[2] Hou P., Fang J., Liu Z., Shi Y., Agostini M., Bernassola F., Bove P., Candi E., Rovella V., Sica G. (2023). Macrophage polarization and metabolism in atherosclerosis. Cell Death Dis..

[3] Khatana C., Saini N.K., Chakrabarti S., Saini V., Sharma A., Saini R.V., Saini A.K. (2020). Mechanistic Insights into the Oxidized Low-Density Lipoprotein-Induced Atherosclerosis. Oxid. Med. Cell Longev..

[4] Linton M.F., Babaev V.R., Huang J., Linton E.F., Tao H., Yancey P.G. (2016). Macrophage apoptosis and efferocytosis in the pathogenesis of atherosclerosis. Circ. J..

[5] Libby P., Hansson G.K. (2019). From Focal Lipid Storage to Systemic Inflammation: JACC Review Topic of the Week. J. Am. Coll. Cardiol..

[6] Dinarello C.A. (2018). Overview of the IL-1 family in innate inflammation and acquired immunity. Immunol. Rev..

[7] Grebe A., Latz E. (2013). Cholesterol crystals and inflammation. Curr. Rheumatol. Rep..

[8] Sharma B.R., Kanneganti T.D. (2021). NLRP3 inflammasome in cancer and metabolic diseases. Nat. Immunol..

[9] Pascual-Figal D.A., Bayes-Genis A., Asensio-Lopez M.C., Hernandez-Vicente A., Garrido-Bravo I., Pastor-Perez F., Diez J., Ibanez B., Lax A. (2019). The Interleukin-1 Axis and Risk of Death in Patients with Acutely Decompensated Heart Failure. J. Am. Coll. Cardiol..

[10] Silvain J., Kerneis M., Zeitouni M., Lattuca B., Galier S., Brugier D., Mertens E., Procopi N., Suc G., Salloum T. (2020). Interleukin-1beta and Risk of Premature Death in Patients with Myocardial Infarction. J. Am. Coll. Cardiol..

[11] Ridker P.M., Everett B.M., Thuren T., MacFadyen J.G., Chang W.H., Ballantyne C., Fonseca F., Nicolau J., Koenig W., Anker S.D. (2017). Antiinflammatory Therapy with Canakinumab for Atherosclerotic Disease. N. Engl. J. Med..

[12] Kattoor A.J., Pothineni N.V.K., Palagiri D., Mehta J.L. (2017). Oxidative stress in atherosclerosis. Curr. Atheroscler. Rep..

[13] Kleinbongard P., Heusch G., Schulz R. (2010). TNFalpha in atherosclerosis, myocardial ischemia/reperfusion and heart failure. Pharmacol. Ther..

[14] de Heredia F.P., Gómez-Martínez S., Marcos A. (2012). Obesity, inflammation and the immune system. Proc. Nutr. Soc..

[15] Alexandraki K., Piperi C., Kalofoutis C., Singh J., Alaveras A., Kalofoutis A. (2006). Inflammatory process in type 2 diabetes: The role of cytokines. Ann. N. Y. Acad. Sci..

[16] Rakipovski G., Rolin B., Nøhr J., Klewe I., Frederiksen K.S., Augustin R., Hecksher-Sørensen J., Ingvorsen C., Polex-Wolf J., Knudsen L.B. (2018). The GLP-1 Analogs Liraglutide and Semaglutide Reduce Atherosclerosis in ApoE-/- and LDLr-/- Mice by a Mechanism That Includes Inflammatory Pathways. JACC Basic Transl. Sci..

[17] Marso S.P., Daniels G.H., Brown-Frandsen K., Kristensen P., Mann J.F., Nauck M.A., Nissen S.E., Pocock S., Poulter N.R., Ravn L.S. (2016). Liraglutide and Cardiovascular Outcomes in Type 2 Diabetes. N. Engl. J. Med..

[18] Husain M., Birkenfeld A.L., Donsmark M., Dungan K., Eliaschewitz F.G., Franco D.R., Jeppesen O.K., Lingvay I., Mosenzon O., Pedersen S.D. (2019). Oral Semaglutide and Cardiovascular Outcomes in Patients with Type 2 Diabetes. N. Engl. J. Med..

[19] Marso S.P., Bain S.C., Consoli A., Eliaschewitz F.G., Jódar E., Leiter L.A., Lingvay I., Rosenstock J., Seufert J., Warren M.L. (2016). Semaglutide and Cardiovascular Outcomes in Patients with Type 2 Diabetes. N. Engl. J. Med..

[20] Gerstein H.C., Colhoun H.M., Dagenais G.R., Diaz R., Lakshmanan M., Pais P., Probstfield J., Riesmeyer J.S., Riddle M.C., Rydén L. (2019). Dulaglutide and cardiovascular outcomes in type 2 diabetes (REWIND): A double-blind, randomised placebo-controlled trial. Lancet.

[21] Marx N., Federici M., Schütt K., Müller-Wieland D., Ajjan R.A., Antunes M.J., Christodorescu R.M., Crawford C., Di Angelantonio E., Eliasson B. (2023). 2023 ESC Guidelines for the management of cardiovascular disease in patients with diabetes. Eur. Heart J..

[22] Brili S., Tousoulis D., Antonopoulos A.S., Antoniades C., Hatzis G., Bakogiannis C., Papageorgiou N., Stefanadis C. (2012). Effects of atorvastatin on endothelial function and the expression of proinflammatory cytokines and adhesion molecules in young subjects with successfully repaired coarctation of aorta. Heart.

[23] Abbasifard M., Kandelouei T., Aslani S., Razi B., Imani D., Fasihi M., Cicero F.G., Sahebkar A. (2022). Effect of statins on the plasma/serum levels of inflammatory markers in patients with cardiovascular disease; a systematic review and meta-analysis of randomized clinical trials. Inflammopharmacology.

[24] Jamialahmadi T., Baratzadeh F., Reiner Ž., Mannarino M.R., Cardenia V., Simental-Mendía L.E., Pirro M., Watts G.F., Sahebkar A. (2022). The Effects of Statin Therapy on Oxidized LDL and Its Antibodies: A Systematic Review and Meta-Analysis. Oxid. Med. Cell. Longev..

[25] Saud A., Ali N., Gali F., Qassam H., Hadi N.R. (2023). The effect of evolocumab alone and in combination with atorvastatin on atherosclerosis progression and TLRs expression. J. Med. Life.

[26] Basiak M., Kosowski M., Hachula M., Okopien B. (2022). Impact of PCSK9 Inhibition on Proinflammatory Cytokines and Matrix Metalloproteinases Release in Patients with Mixed Hyperlipidemia and Vulnerable Atherosclerotic Plaque. Pharmaceuticals.

[27] Yang J., Ma X., Niu D., Sun Y., Chai X., Deng Y., Wang J., Dong J. (2023). PCSK9 inhibitors suppress oxidative stress and inflammation in atherosclerotic development by promoting macrophage autophagy. Am. J. Transl. Res..

[28] Que Q., Guo X., Zhan L., Chen S., Zhang Z., Ni X., Ye B., Wan S. (2019). The GLP-1 agonist, liraglutide, ameliorates inflammation through the activation of the PKA/CREB pathway in a rat model of knee osteoarthritis. J Inflamm (Lond.).

[29] Tao Y., Ge G., Wang Q., Wang W., Zhang W., Bai J., Lin J., Shen J., Guo X., Xu Y. (2019). Exenatide ameliorates inflammatory response in human rheumatoid arthritis fibroblast-like synoviocytes. IUBMB Life.

[30] Ferdaoussi M., Abdelli S., Yang J.Y., Cornu M., Niederhauser G., Favre D., Widmann C., Regazzi R., Thorens B., Waeber G. (2008). Exendin-4 protects beta-cells from interleukin-1 beta-induced apoptosis by interfering with the c-Jun NH2-terminal kinase pathway. Diabetes.

[31] Wang Y., Parlevliet E.T., Geerling J.J., van der Tuin S.J., Zhang H., Bieghs V., Jawad A.H., Shiri-Sverdlov R., Bot I., de Jager S.C. (2014). Exendin-4 decreases liver inflammation and atherosclerosis development simultaneously by reducing macrophage infiltration. Br. J. Pharmacol..

[32] Dai Y., Dai D., Wang X., Ding Z., Li C., Mehta J.L. (2014). GLP-1 agonists inhibit ox-LDL uptake in macrophages by activating protein kinase A. J. Cardiovasc. Pharmacol..

[33] Tanaka M., Matsuo Y., Yamakage H., Masuda S., Terada Y., Muranaka K., Wada H., Hasegawa K., Shimatsu A., Satoh-Asahara N. (2016). Differential effects of GLP-1 receptor agonist on foam cell formation in monocytes between non-obese and obese subjects. Metabolism.

[34] Ma G.F., Chen S., Yin L., Gao X.D., Yao W.B. (2014). Exendin-4 ameliorates oxidized-LDL-induced inhibition of macrophage migration in vitro via the NF-κB pathway. Acta Pharmacol. Sin..

[35] Zobel E.H., Ripa R.S., von Scholten B.J., Curovic V.R., Diaz L.J., Hansen T.W., Rossing P., Kjaer A. (2021). Effect of Liraglutide on Vascular Inflammation Evaluated by [64Cu]DOTATATE. Diagnostics.

[36] Luna-Marco C., de Marañon A.M., Hermo-Argibay A., Rodriguez-Hernandez Y., Hermenejildo J., Fernandez-Reyes M., Apostolova N., Vila J., Sola E., Morillas C. (2023). Effects of GLP-1 receptor agonists on mitochondrial function, inflammatory markers and leukocyte-endothelium interactions in type 2 diabetes. Redox Biol..

[37] Hogan A.E., Gaoatswe G., Lynch L., Corrigan M.A., Woods C., O’Connell J., O’Shea D. (2014). Glucagon-like peptide 1 analogue therapy directly modulates innate immune-mediated inflammation in in-dividuals with type 2 diabetes mellitus. Diabetologia.

[38] Belančić A., Kresović A., Troskot Dijan M. (2021). Glucagon-like peptide-1 receptor agonists in the era of COVID-19: Friend or foe?. Clin. Obes..

